# Over-Activated Proteasome Mediates Neuroinflammation on Acute Intracerebral Hemorrhage in Rats

**DOI:** 10.3390/cells8111326

**Published:** 2019-10-27

**Authors:** Hock-Kean Liew, Wei-Fen Hu, Peter Bor-Chian Lin, Po-Kai Wang, Andy Po-Yi Tsai, Cheng-Yoong Pang, Tsung-Ying Chen

**Affiliations:** 1 Department of Medical Research, Hualien Tzu Chi Hospital, Buddhist Tzu Chi Medical Foundation, Hualien 970, Taiwan; hockkean@tzuchi.com.tw (H.-K.L.); 103327105@gms.tcu.edu.tw (W.-F.H.); 2 Neuro-Medical Scientific Center, Hualien Tzu Chi Hospital, Buddhist Tzu Chi Medical Foundation, Hualien 970, Taiwan; 3 Cardiovascular and Metabolomics Research Center, Hualien Tzu Chi Hospital, Buddhist Tzu Chi Medical Foundation, Hualien 970, Taiwan; 4 PhD Program in Pharmacology and Toxicology, Tzu Chi University, Hualien 970, Taiwan; 5 Indiana University School of Medicine, Indianapolis, IN 46202, USA; roc800703@gmail.com (P.B.-C.L.); boypoyi@gmail.com (A.P.-Y.T.); 6 Department of Anesthesiology, Hualien Tzu Chi Hospital, Buddhist Tzu Chi Medical Foundation, Hualien 970, Taiwan; 7 School of Medicine, Tzu Chi University, Hualien 970, Taiwan; 8 Department of Medical Education, Hualien Tzu Chi Hospital, Buddhist Tzu Chi Medical Foundation, Hualien 970, Taiwan

**Keywords:** ER stress, GRP78, intracerebral hemorrhage, NFκB, oxidative stress, proteasome activity, proteostasis disturbance, protein aggregation, ubiquitination

## Abstract

Background: Neuroinflammation is a hallmark in intracerebral hemorrhage (ICH) that induces secondary brain injury, leading to neuronal cell death. ER stress-triggered apoptosis and proteostasis disruption caused neuroinflammation to play an important role in various neurological disorders. The consequences of ER stress and proteostasis disruption have rarely been studied during the course of ICH development. Methods: ICH was induced by collagenase VII-S intrastriatal infusion. Animals were sacrificed at 0, 3, 6, 24, and 72 h post-ICH. Rats were determined for body weight changes, hematoma volume, and neurological deficits. Brain tissues were harvested for molecular signaling analysis either for ELISA, immunoblotting, immunoprecipitation, RT-qPCR, protein aggregation, or for histological examination. A non-selective proteasome inhibitor, MG132, was administered into the right striatum three hours prior to ICH induction. Results: ICH-induced acute proteasome over-activation caused the early degradation of the endoplasmic reticulum (ER) chaperone GRP78 and IκB protein. These exacerbations were accompanied by the elevation of pro-apoptotic CCAAT-enhancer-binding protein homologous protein (CHOP) and pro-inflammatory cytokines expression via nuclear factor-kappa B (NF-κB) signal activation. Pre-treatment with proteasome inhibitor MG132 significantly ameliorated the ICH-induced ER stress/proteostasis disruption, pro-inflammatory cytokines, neuronal cells apoptosis, and neurological deficits. Conclusions: ICH induced rapid proteasome over-activation, leading to an exaggeration of the ER stress/proteostasis disruption, and neuroinflammation might be a critical event in acute ICH pathology.

## 1. Introduction

Bleeding in the brain parenchyma or spontaneous intracerebral hemorrhage (ICH) accounts for approximately 10% to 15% of all cerebral strokes, and has an incidence of 4.3 per 10,000 person-years [1]. The pathology of ICH is primarily due to mechanical damages associated with the progression of the growing hematoma mass, which occurs most frequently in the putamen and dorsal striatum [1]. Hematoma expansion causes a mass effect that leads to local compression and breakdown of the microvasculature, consequently disturbing the cerebral blood flow [1,2,3,4]. The secondary injury of the ICH is caused by a coagulation–hemolysis–hemoglobin breakdown cascade [5]. The hemolysis of the hematoma occurs from hours to a few days after ICH [6,7,8], producing hemolysate that contains hemoglobin as well as its breakdown products, i.e., iron, heme, and degraded heme products. These products increase reactive oxygen species (ROS)-related oxidative stress [8,9,10] that causes damages to DNA, protein, and lipids [11,12], resulting in neuroinflammation and delayed edema formation in the brain [9,13,14]. 

The endoplasmic reticulum (ER) is a major intracellular organelle that facilitates protein translocation, folding, and post-translational modifications. Misfolded or immature proteins aggregate in the ER lumen and trigger an adaptive program known as the unfolded protein response (UPR) [15]. The accumulation of misfolded proteins in the ER prompts the cell to initiate UPR to restore protein homeostasis (also known as proteostasis) [15,16]. Under normal circumstances, misfolded or harmful proteins are ubiquitinated and degraded by the proteasome; however, different perturbations at the cellular level can affect ER homeostasis and thus impair UPR function, resulting in a pathological state of ER stress [16,17]. During ER stress, UPR helps to remove misfolded proteins by regulating ER-associated protein degradation (ERAD). ERAD is a major intracellular mechanism that works to prevent the accumulation of misfolded proteins that might lead to toxic protein aggregation and subsequent cell death. Previous studies demonstrated that excessive ER stress could trigger apoptotic cascade, and plays a vital role in a range of neurological disorders including Huntington’s disease, Parkinson’s disease, spinal cord injury, Alzheimer’s disease, and cerebral ischemia [16,18]. 

During ICH, cell metabolism is disrupted and a series of stress responses are activated [19]. The UPR consists of three main signaling systems that are initiated by three ER membrane-associated sensors: protein kinase RNA-like endoplasmic reticulum kinase (PERK), inositol-requiring enzyme-1α (IRE-1α), and activating transcription factor 6 (ATF6) [20,21,22]. These three sensors are all associated with glucose-regulated protein 78 kDa (GRP78, a key ER chaperone that is also known as BiP), which controls their activation through a binding/release mechanism. Ordinarily, the activation of PERK, IRE-1α, and ATF6 are prevented by the binding of GRP78 [20,23,24,25]. However, during unresolvable ER stress conditions, the UPR fails to reduce ER stress and restore homeostasis, promoting cell death [20,22].

Proteasomes has been reported to activate NF-κB through degradation of the inhibitor of κB (IкB), which in turn induced inflammation [26]. Several studies demonstrated that proteasome inhibitors such as MLN519, CVT-634, MG132, or bortezomib can reduce post-stroke inflammation and protect against cerebral injury [27]. However, the effect of proteasome inhibition on hemorrhagic strokes has rarely been investigated. Therefore, this study aimed to clarify the effects of ER dysfunction and proteostasis disturbance in ICH rats.

## 2. Experimental Section

### 2.1. Methods

#### 2.1.1. Animals 

A total of 108 eight-week-old adult male Sprague–Dawley (SD) rats weighing 300–350 g were included in this study. All animal experiment protocols were approved by the Institutional Animal Care and Use Committee (IUCAC) Hualien Tzu Chi Hospital (Approval No. IUCAC 103-40), Taiwan, following guidelines set by the National Institute of Health Guide for the Care and Use of Laboratory Animals. Animals were housed under a 12-h light/12-h dark cycle with free access to food and water. Utmost efforts were made to minimize the suffering and the number of animals used. 

#### 2.1.2. Intracerebral Hemorrhage Induction Model

The animals were anesthetized with intraperitoneal injection of pentobarbital (50 mg/kg). ICH was induced by stereotaxic infusion of bacterial collagenase VII-S (0.2 U in 1.0-μL sterile saline, Sigma-Aldrich, St. Louis, MO, USA) with the infusion rate 0.2 μL/min [28,29]. To create a more severe hemorrhagic condition, a 0.6 U in 3.0-μL sterile saline of bacterial collagenase VII-S was infused into the right striatum. An additional model, the blood-infusion model, was also used as a comparison. One hundred microliter of autologous blood (AB) drawn from the tail vein was immediately infused into the right striatum of the experimental rat. The needle was kept in place for 10 min to prevent backflow.

#### 2.1.3. The Proteasome Inhibitors Administration

A non-selective proteasome inhibitor: MG132 (0.42 μg in 2 μL 5% DMSO-sterile saline per rat, cat.474790, Merck, Darmstadt, Germany) was microinjected into the right striatum of the rat (0.0 mm posterior, 3.0 mm right, 5.0 mm ventral to bregma at the skull surface) 3 h prior to ICH induction.

### 2.2. Morphometric Measurements

A morphometric measurement was used to determine the hematoma volume as described previously [28,29]. Rat brains were removed and cut coronally through the needle entry plane (identified on the brain surface), and then serially sliced (2-mm thickness) anteriorly and posteriorly to the needle entry site. Images of serial slices were taken by a digital camera and quantitated with Image J (NIH, Bethesda, MA, USA). Digital photographs of the serial slices with blood clots were obtained, and total hematoma volume (mm^3^) was measured by multiplying each section by the distance of sections containing blood clots. 

### 2.3. Neurobehavioral Assays

The modified Neurological Severity Score (mNSS) scale was used to evaluate the neurological abnormalities prior to ICH injury and at 24 and 72 h after ICH. The mNSS study was carried out in blinded manner by two well-trained investigators. The mNSS comprises tests of motor, sensory, and balance functions. Neurological function is graded from 0–18 points (normal score, 0; maximal deficit score, 18). The higher the scores, the more severe the neurological deficits [30]. 

### 2.4. Protein Carbonyl Assay 

Due to the relatively early formation and stability compared with other oxidative products, protein carbonyl groups were observed in several diseases as an immediate and high-quality set biomarkers of oxidative stress [31]. 2,4-dinitrophenylhydrazine (DNPH) tagging of protein carbonyls is one of the most common ways of measuring oxidative stress. The level of protein carbonyls in striatal lysates was measured according to the manufacturer’s directions (Protein Carbonyl Assay Kit, cat. ab126287; Abcam, Cambridge, MA, USA). After the extraction of protein from striatal tissues, protein concentration was then determined by Bio-Rad Protein Assay (Cat. 500-0006; Bio-Rad, Hercules, CA, USA). A total of 1 mg of striatal protein lysates was assessed in the measurement of protein carbonyl contents. DNPH was added to react with striatal protein carbonyls, forming a Schiff base to produce hydrazone, which was determined by absorbance at 375 nm. 

### 2.5. Ubiquitin Competitive Enzyme-Linked Immunosorbent Assay

Ubiquitin levels have been used as a marker of ER stress in various types of diseases [32,33]. We measured the content of ubiquitinated protein in the striatal lysates to evaluate the level of ER stress after ICH injury. The measurement of ubiquitinated protein was performed by competitive enzyme-linked immunosorbent assay (ELISA) kits (abx576016; Abbexa, Cambridge, UK) according to the manufacturer’s instructions. In brief, biotin standards and striatal lysates were added to the ubiquitin-specific antibody pre-coated 96-well plate, and detection was performed by adding another biotin-conjugated anti-Ubiquitin antibody. Avidin conjugated to horseradish peroxidase (HRP) was added to each well, and 3,3′,5,5′-tetramethylbenzidine substrate was added to measure the activity of the bound HRP in each well. The final chromogen was stabilized by adding acidic stop solution, and absorbance at 450 nm was measured spectrophotometrically with a microplate reader; then, the concentration of ubiquitin was calculated.

### 2.6. Cytokines Assay

A total of 50 μg of the ipsilateral striatal protein at 3, 24, and 72 h post-ICH were used to estimate the level of pro-inflammatory cytokines: Tumor necrosis factor-α (TNF-α), interleukin-1β (IL-1β), interleukin-6 (IL-6), and anti-inflammatory cytokine: interleukin-10 (IL-10) in the ipsilateral striatum lysate were estimated using ELISA kits (TNF-a Rat ELISA kit # DY510, IL-1β Rat ELISA kit # DY501, IL-6 Rat ELISA kit # DY506, IL-10 Rat ELISA kit # DY522, R & D Systems, Minneapolis, MN, USA, respectively) according to the manufacturer’s instructions. 

### 2.7. Proteasome Activity Assay

The proteasome activity assay was performed using a fluorometric assay kit (Proteasome Activity Fluorometric Assay Kit, cat. K245; Biovision, Milpitas, CA, USA) in striatal lysates at 3, 24, and 72 h post-ICH according to the manufacturer’s directions. Briefly, 100 µg of fresh striatal protein lysates were first incubated in a 96-well plate at 37 °C for 60 min with a succinyl-LLVY-7-Amino-4-methylcoumarin (AMC) substrate. Striatal lysates co-incubate with succinyl-LLVY-AMC and MG-132, which suppresses proteasome-related proteolytic activity, and represents a non-proteasome activity. The amount of fluorescent AMC was measured with a spectrometer at a wavelength of 440 nm, with an excitation wavelength at 380 nm [34].

### 2.8. Protein Aggregation Assay

An equal amount of protein (40 μg) in striatal lysates was denatured by the incubation in loading buffer without dithiothreitol (DTT). The denatured samples were processed through electrophoresis and blotting onto a polyvinylidene difluoride (PVDF) membrane. The primary antibody is anti-ubiquitin (ab52664; Abcam, Cambridge, UK, 1:2000) followed by secondary antibody (AP308P; EMD Millipore, Billerica, MA, USA, 1:20000), and finally detected by a chemiluminescent ECL Plus Western blotting detection system (RPN2133; Amersham Biosciences, Little Chalfont, UK). Intensities of bands/lanes (polyubiquitinated protein with molecular weight >50 kDa) [35] were quantified with a densitometry analysis system (GS-800 Calibrated Densitometer, Bio-Rad, Hercules, CA, USA) and calculated as the optical density × area. 

### 2.9. Immunoprecipitation and Immunoblotting

Proteins were immuneprecipitated via incubation of the tissue homogenate lysates overnight at 4 °C with anti-GRP78 antibody (1:1000, Abcam, Cambridge, MA, USA) or anti-IκB antibody (1:50, Cell Signaling Technology, Danvers, MA, USA), followed by the addition of protein A/G–Magnetic beads (Thermo Fisher Scientific, Waltham, MA, USA) and incubation for 1 h at 25 °C. The beads were washed four times with lysis buffer and were boiled in 20 μL SDS loading buffer. The total cellular lysates (20 μg) were separated by 10% SDS-PAGE and transferred onto a PVDF membrane (Millipore, Billerica, MA, USA). The blots were incubated with the following specific antibodies: anti-ubiquitin (1:5000, Abcam, Cambridge MA) and anti-β-actin (1:20000, Sigma-Aldrich, St. Louis, MO, USA) for the immunoprecipitated experiment. For the common immunoblotting, the specific antibodies: anti-GRP78, anti-CHOP (1:1000, Santa-Cruz, Dallas, TX, USA), anti-phospho-NF-κB (Ser536) (1:500, Cell Signaling Technology), anti-α-tubulin (1:1000, GeneTex, Irvine, CA, USA), and anti-IκB (1:1000, Cell Signaling Technology) were used to detect the expression with the ipsilateral and contralateral ICH striatal lysates. Then, blots were incubated with appropriate HRP-conjugated secondary antibodies, and the proteins were detected using the Western Lightning Plus-ECL (PerkinElmer, Waltham, MA, USA). The chemiluminescence was visualized using Amersham Hyperfilm ECL film (GE Healthcare, Buckinghamshire, UK).

### 2.10. Reverse Transcription Quantitative Real-Time Polymerase Chain Reaction (RT-qPCR)

Total RNA was extracted from rat striatum using the Total RNA Isolation Kit (tissue, GeneDireX, Taipei, Taiwan). The cDNA was synthesized using the SuperScript^®^ III First-Strand Synthesis System for RT-PCR (Invitrogen, Carlsbad, CA, USA). The real-time PCR primers used in this study included: GRP78 forward: AACCAAGGATGCTGGCACTA, reverse: ATGACCCGCTGATCAAAGTC; CHOP forward: GGAAGTGCATCTTCATACACCACC, reverse: TGACTGGAATCTGGAGAGAGCGAGGGC; XBP1 forward: AGCAGGTGCAGGCCCAGTT, reverse: TAGCAGACTCTGGGGAAGGA; sXBP1 forward: TGCTGAGTCCGCAGCAGGTG, reverse: GCTGGCAGGCTCTGGGGAAG; ATF4 forward: CCTTCGACCAGTCGGGTTTG, reverse: CTGTCCCGGAAAAGGCATCC. Quantitative real-time PCR was performed in a reaction mixture containing cDNA, 200 nM of specific primers, and Maxima SYBR Green/ROX qPCR Master Mix (Fermentas, Waltham, MA, USA). PCR amplification was performed in a QuantStudio 5 Real-Time PCR System (Applied Biosystems, Waltham, MA, USA). The following PCR conditions were used: 50 °C for 2 min, 95 °C for 10 min, 40 cycles of 95 °C for 15 s, 60 °C for 1 min, and 60 °C for 30 s. The ΔΔCt method was used for data analysis [36]. The mRNA expression levels were measured in triplicate and normalized to the expression levels of β-actin in the same samples. 

### 2.11. Immunohistochemical Staining 

Under deep anesthesia, rats were transcardially perfused through the left ventricle with saline and followed by 4% paraformaldehyde. Brains were removed and then post-fixed in 4% paraformaldehyde at room temperature for 2 h, cryoprotected in 30% (w/v) sucrose (4 °C), embedded in Tissue-Tek O.C.T. compound, frozen, and stored at −80 °C until further analysis. Serial coronal sections (20-μm thickness) were cut on a freezing sliding microtome. Tissue sections were blocked with 3% donkey normal serum and 2% bovine serum albumin (BSA) in PBS and incubated overnight with primary antibodies. The following antibodies were used: anti-GFAP (1:500, NOVUS, Centennial, CO, USA), anti-NeuN (1:1000, Millipore, Billerica, MA, USA), anti-RECA (1:200, Abcam, Cambridge, MA, USA), anti-OX-42 (1:200, Bio-Rad), anti-ubiquitin (1:200, Abcam, Cambridge, MA, USA) anti-GRP78 (1:50, Cell Signaling Technology), and anti-CHOP (1:50, Santa-Cruz). After washing, the respective secondary antibody conjugated with fluorescein (FITC) or rhodamine (Jackson ImmunoResearch, West Grove. PA, USA) was applied to sections for 1 h at room temperature. Then, sections were washed and mounted with 50% glycerol in PBS. Samples were examined with a Zeiss Axiovert 200 M fluorescent microscope or a Zeiss LSM 510 META confocal imaging system (Carl Zeiss, Oberkochen, Germany). 

### 2.12. TUNEL Assay

Terminal deoxynucleotidyl transferase dUTP nick end labeling (TUNEL) staining was performed using an In Situ Cell Death Detection Kit, POD (Roche, Basel, Switzerland) according to the manufacturer’s instructions. The slices were collected at +1.0, 0.0, and −1.0 mm (respective to the center of the hemorrhagic lesion) anterior and posterior to bregma using a cryostat (CM 1900, Leica, Wetzlar, Germany). After fixing and permeabilizing, TUNEL-positive cells were detected by incubating with TUNEL reaction mixture for 1 h at 37 °C. TUNEL signal was detected with FITC-labeled secondary antibody for streptavidin. Cell nuclei were counterstained with DAPI. Negative controls of TUNEL staining were performed by omitting terminal deoxynucleotidyl transferase (TdT). The number of apoptotic/necrotic cells was determined by counting the TUNEL-positive cells/DAPI cells in the four specific regions around the hematoma core. For double staining, sections were subjected to heat-induced epitope retrieval and incubated with the following specific antibodies (NeuN, GFAP, OX-42, RECA, ubiquitin, GRP78, and CHOP), respectively. After specific antibody detection with secondary antibody, sections were processed for TUNEL staining. 

### 2.13. Transmission Electron Microscopy

Transmission electron microscopy (TEM) was performed using a Hitachi H-7500 transmission electron microscope (Electron Microscopy Laboratory, Tzu Chi University, Hualien, Taiwan) equipped with an AMT XR-16 16mp high-resolution charge-coupled device camera and operating at 80 kV to explore the morphology, size, and distribution of aggregates protein in 3 h post-ICH injury. Small blocks of striatal tissue were pre-fixed with 2.5% glutaraldehyde prepared in 0.1 M cacodylate buffer containing 2% tannic acid at 0–4°C for 1 h, and post-fixed with 1% OsO4 in 0.1 M cacodylate buffer for 1 h at room temperature. After fixation, specimens were dehydrated through a graded series of ethanol and embedded in Spurr’s resin. Serial ultrathin sections of approximately 80 nm were made with a Leica Ultra cut R ultramicrotome (Leica, Heerbrugg, Switzerland) and stained with uranyl acetate and lead citrate. Images were analyzed using the AMT Capture Engine, v602.600.51. 

### 2.14. Statistical Analysis 

Data were statistically analyzed using Prism software (GraphPad Software, San Diego, CA, USA) for Student’s t-test, and are presented as mean ± standard error mean (S.E.M.). The statistical comparisons among multiple groups were made using one-way ANOVA, and multiple time points by two-way ANOVA were followed by Bonferroni correction. In all instances, n refers to the number of animals in a particular group. A *p* value of < 0.05 is considered statistically significant.

## 3. Results 

### 3.1. ICH Increases Oxidative Stress, Hematoma Expansion, Body Weight Loss, and Neurological Deficits 

**Figure 1:**
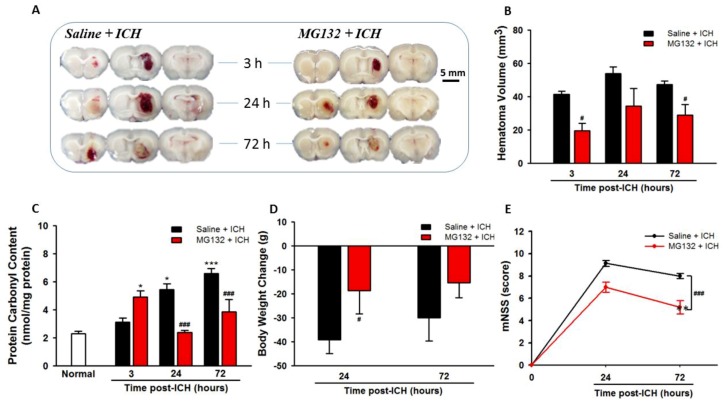
MG132 pre-treatment reduced hematoma volume, oxidative stress, body weight loss, and neurological impairments of intracerebral hemorrhage (ICH) rats. Representative image of hemorrhagic brain sections in ICH and MG132 pre-treated-ICH rats at 3, 24, and 72 h post-ICH injury (**A**). Statistical hematoma expansion volume (**B**), protein carbonyl content (**C**), body weight change (**D**), and modified Neurological Severity Score (mNSS) neurobehavioral assay (**E**) at 0 (normal), 3, 24, and 72 h post-ICH; respectively. Values are indicated by means ± SEM; n = 6 each group, * *p* < 0.05; *** *p* < 0.001; compared to the normal group; ^#^ *p* < 0.05; ^###^ *p* < 0.001; compared to the Saline + ICH group, respectively.

Oxidative stress plays a pivotal role in the pathogenesis of ICH [7,11,12,37,38]. To identify the oxidative stress in the ICH brain, we measured the protein carbonyl contents at 0 (normal), 3, 24, and 72 h post-ICH. In comparison to normal striatal tissue, protein carbonyl content assay revealed that all rats suffering from ICH injury showed significantly elevated levels of protein carbonyl in the ipsilateral striatal tissue at 24 h (Figure 1C, *p* < 0.05) and 72 h (Figure 1C, *p* < 0.001), respectively, as well as the volume of the hematoma (Figure 1B). The loss of body weight (Figure 1D) and the neurological deficits (Figure 1E) were significantly increased at 24 h and 72 h post-ICH, respectively.

### 3.2. ICH Induces ER Stress and Proteostasis Disruption

**Figure 2:**
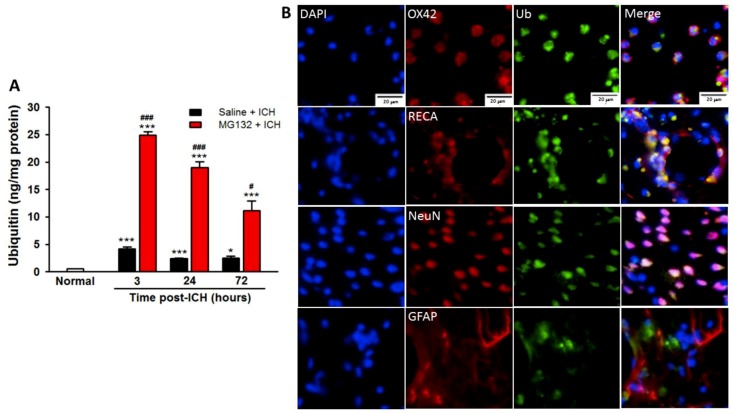
Expression of ubiquitinated protein in ICH injury striatum. Ubiquitin protein levels evaluated by ubiquitin competitive enzyme-linked immunosorbent assay at normal, 3, 24, and 72 h post-ICH injury (**A**). Rat brains were stained for ubiquitin (green) and dual stained with OX-42 (anti-CD11b/c; microglia, red), RECA (anti-rat endothelial cells antigen; vascular endothelial cells, red), NeuN (anti-neuronal nuclei; neuron, red) and GFAP (anti-glial fibrillary acidic protein; astrocyte, red) in the ipsilateral striatal (3 h post-ICH) (**B**). Value are indicated by means ± SEM; n = 6 each group, * *p* < 0.05; *** *p* < 0.001; compared to normal group; ^#^ *p* < 0.05; ^###^ *p* < 0.001; compared to Saline + ICH group, respectively.

**Figure 3:**
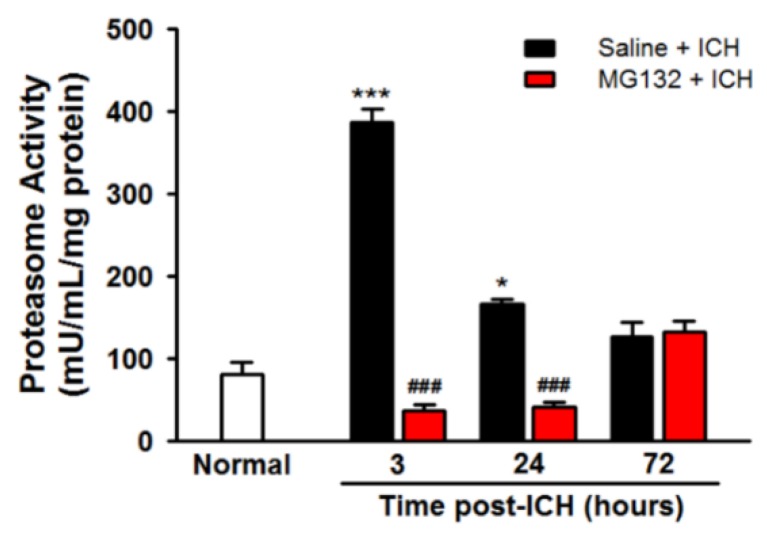
ICH induced acute proteasome over-activation. The proteasome activity was measured by fluorometric assay in ipsilateral striatal lysates at normal, 3, 24, and 72 h post-ICH. Values are indicated by means ± SEM; n = 6 each group, * *p* < 0.05; *** *p* < 0.001; compared to the normal group; ^###^ *p* < 0.001; compared to the Saline + ICH group, respectively.

**Figure 4:**
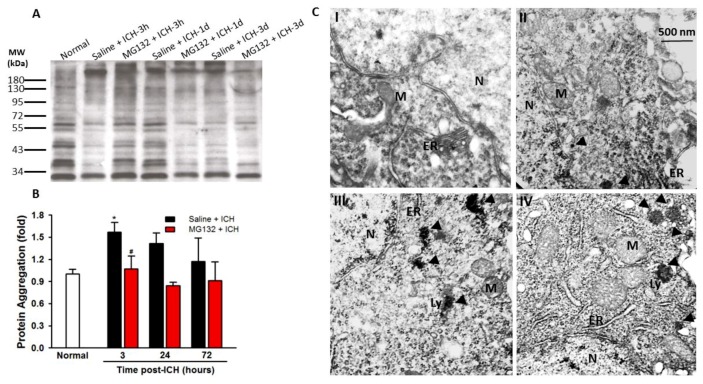
Ubiquitinated protein aggregates in ICH striatal. Representative naïve SDS-PAGE of ubiquitinylated protein accumulation (**A**) and statistical analysis of high molecular weight (>50 kDa) polyubiqutinated protein aggregation in normal, 3, 24, 72 h post-ICH injury, n = 6 each group (**B**). Transmission electron microscopic pictures of neuronal stroma stained with uranyl acetate and lead citrate in region of contralateral striatum (I), perihematomal striatal (II and III) and core (IV) at 3 h post-ICH. ER, endoplasmic reticulum; Ly, lysosome; M, mitochondria; N, nucleus; abnormal protein aggregated (arrow point) (**C**). Values are indicated by means ± SEM; * *p* < 0.05; compared to the normal group; ^#^ *p* < 0.05; compared to the Saline + ICH group, respectively (**B**).

To investigate the influence of the ubiquitin/proteasome pathway in ICH, we evaluated the levels of ubiquitinated protein levels (ubiquitin protein accumulation served as a specific marker of misfolded/unfolded protein) (Figure 2), proteasome activity (Figure 3), and protein aggregation (Figure 4), respectively. Our results demonstrated that misfolded/unfolded protein (ubiquitinated protein, Figure 2A) accumulated as early as 3 h post-ICH (*p* < 0.001 as compared with normal control), and sustained to 72 h post-ICH (*p* < 0.05 as compared with normal control), respectively. The misfolded/unfolded protein (Ub-positive cells) is majorly localized around the perihematomal area and co-localized with microglia (OX-42, Figure 2B), vascular endothelial cells (RECA, Figure 2B), and neurons (NeuN, Figure 2B), but not astrocytes (GFAP, Figure 2B). 

### 3.3. ICH Induces Proteasome Over-Activation

ICH significantly induced the proteasome activity (four-fold increased as compared with normal control at 3 h post-ICH, *p* < 0.001, Figure 3) without affecting the constitutive proteasome protein (data not shown). In order to cope with the ICH-induced massive misfolded/unfolded protein, ICH injury induced an over-activated proteasome; however, this upregulated proteasome activity failed to fully cope with the ICH-induced misfolded/unfolded protein in the hemorrhagic striatum, leading to protein aggregated (polyubiquitinated with high molecular weight >50 kDa) at 3 h (Figure 4A,B, *p* < 0.05). The ICH-induced early protein aggregation was further confirmed by TEM (Figure 4C) at 3 h post-ICH ipsilateral striatum. 

### 3.4. GRP78 Protein Degradation Occurs at the Early Phase Of ICH and Coincides with the Activation Of Pro-Apoptotic Transcriptional Factor CHOP

**Figure 5:**
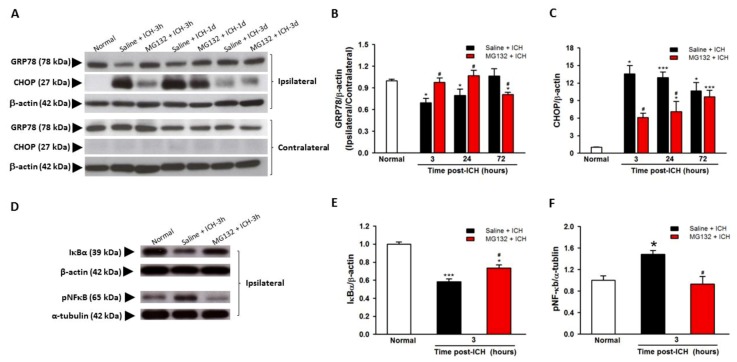
Effects of MG132 pretreatment on GRP78, CHOP, IκB, and pNF-κB protein levels after ICH injury. Representative Western blotting images showing the 78-kDa band of GRP78, 19-kDa band of CHOP (**A**), and pNF-κB, and IκB (**D**). Quantitative analyses of GRP78 (**B**) and CHOP (**C**) protein detected in the ipsilateral and contralateral striatum lysate from normal, Saline + ICH-3h, MG132 + ICH-3h, Saline + ICH-24 h, MG132 + ICH-24h, Saline + ICH-72h, and MG132 + ICH-72h animals, respectively. Quantitative analyses of IκB (**E**) and pNF-κB (**F**) protein detected in the ipsilateral striatum lysate from normal, Saline + ICH-3h, and MG132 + ICH-3h animals, respectively. Values are indicated by means ± SEM; n = 6 each group, * *p* < 0.05; *** *p* < 0.001; compared to the normal group; ^#^ *p* < 0.05; compared to the Saline + ICH group, respectively.

**Figure 6:**
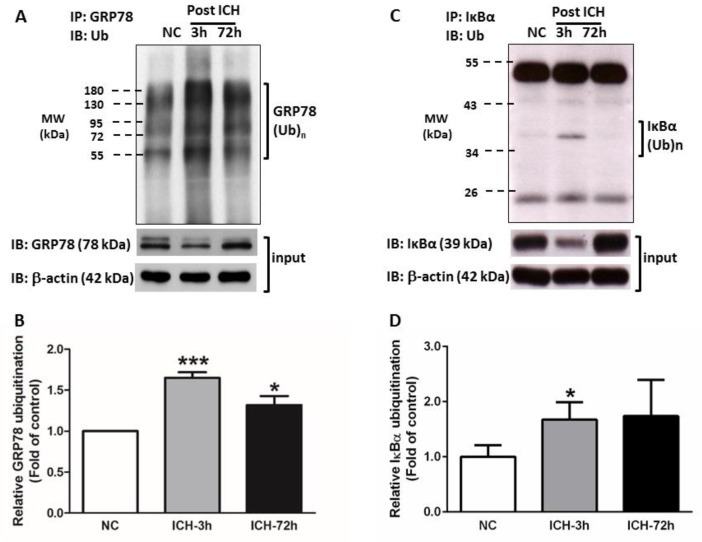
ICH induced the protein degradation of GRP78 and IκB protein. Representative Western blotting analysis of striatal tissue lysates immunoprecipitated with anti-glucose-regulated protein 78 kDa (GRP78) (**A**) and anti-IκB (**C**) antibodies followed by anti-ubiquitin antibody at normal (NC), 3 (ICH-3h), and 72 h (ICH-72h) post-ICH injury. Value of quantitative of relative ubiquitinated GRP78 (**B**) and IκB (**D**) are indicated by means ± SEM; n = 4 each group, * *p* < 0.05; *** *p* < 0.001; compared to the normal group, respectively.

**Figure 7:**
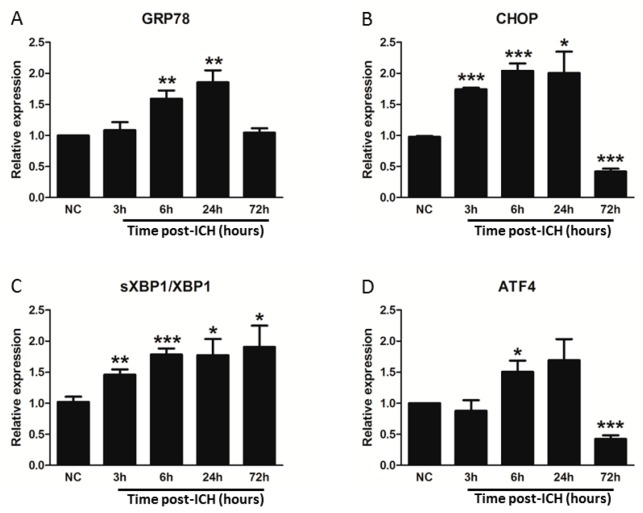
The mRNA expression of GRP78, CHOP, sXBP1/XBP1 ratio, and ATF4 in ipsilateral striatum. The RT-qPCR measured the mRNA levels for GRP78 (**A**), CHOP (**B**), sXBP1/XBP1 (**C**), and ATF4 (**D**) in the ipsilateral striatum at normal control (NC), 3 h, 6 h, 24 h, and 72 h post-ICH injury. Values are indicated by means ± SEM; n = 6 each group, * *p* < 0.05; ** *p* < 0.01; *** *p* < 0.001; compared to the normal control group, respectively.

The chaperone GRP78 protein is an important responder for ER stress. Our immunoblotting experiment revealed that in normal or the contralateral striatal tissues, the expression of chaperone GRP78 protein was not affected by ICH injury, but significantly reduced at 3 h after ICH (*p* < 0.05) and started to restore at 72 h (Figure 5A,B) in the ipsilateral striatum. The RT-qPCR analysis demonstrated that GRP78 mRNA was elevated at 6 (*p* < 0.01) and 24 h (*p* < 0.01), respectively, after ICH insult (Figure 6A). At 72 h post-ICH, the transcription of GRP78 was identical to the normal striatum (Figure 7A). In the identification of immunoprecipitated GRP78 with anti-ubiquitin antibody, we demonstrated an elevation of ubiquitinated GRP78 at the 3 h (Figure 6A,B, *p* < 0.001) and 72 h (Figure 6A,B, *p* < 0.05). Taken together, the downregulation of GRP78 was not due to the decrease of the GRP78 biogenesis during the acute phase of ICH injury. Instead, it is caused by an increased with the ubiquitin-proteasome degradation of the GRP78 protein. Besides, the striatal GRP78 reduction was significantly more drastic in an 0.6 U collagenase-ICH model (I_3_, *p* < 0.001 versus normal striatal) at 3 h post-ICH (Figure S1E,F). The decline of GRP78 protein was also noted in the autologous blood (AB) infusion ICH model (*p* < 0.05), which is a less severe type of ICH induction model (Figure S1E,F). The downregulation of chaperone GRP78 was correlated with the severity of hematoma volume; the bigger the hematoma volume, the much more decreased the GRP78 protein (Figure S1F).

**Figure 8:**
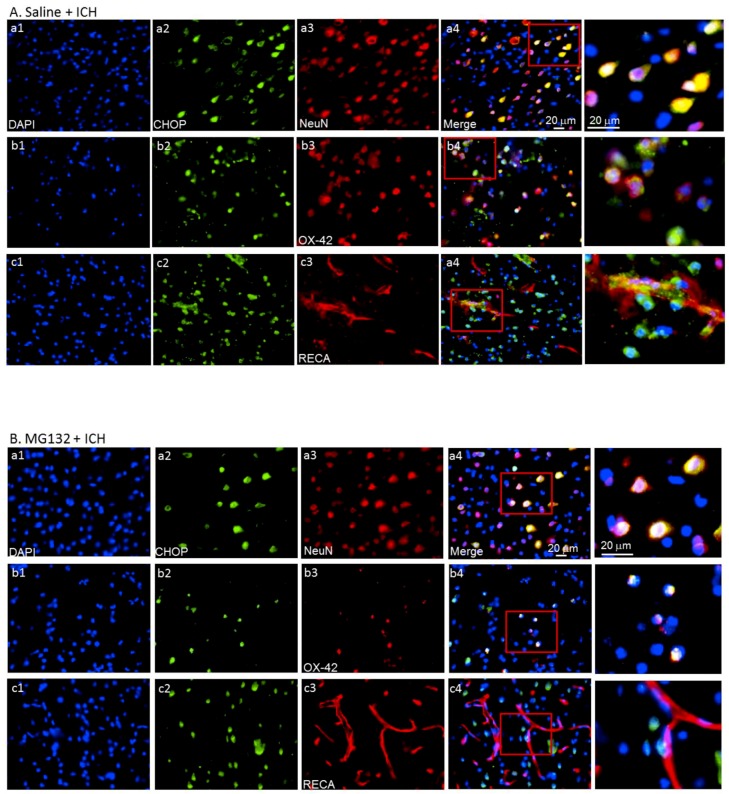
Immunohistochemical staining of CHOP expression at 1 day post-ICH. Yellowish color in the merges (a4, b4, and c4) indicates the co-localization of CHOP expression in neurons (NeuN, a1–a5), microglias (OX-42, b1–b5), and endothelial cells (RECA, c1–c5) in Saline + ICH (**A**) and MG132 + ICH (**B**), respectively.

In parallel, the immunoblotting results demonstrated that the CHOP was significantly detected and elevated as early as 3 h (*p* < 0.05) and lasted for 72 h (*p* < 0.05) in ipsilateral hemorrhagic striatum after ICH (Figure 5D). Meanwhile, the pro-apoptotic CHOP protein was not detected in the normal striatum of healthy rats or contralateral striatum of ICH rats at 3-h, 6-h, 24-h, and 72-h intervals post-ICH (Figure 5A), respectively. Besides, the transcription of pro-apoptotic CHOP mRNA is induced as early as at 3 h post-ICH (Figure 5B, *p* < 0.001) and decreased at 72 h after ICH injury (Figure 5B, *p* < 0.001). The increased CHOP protein expression after ICH injury majorly localized in the neurons (Figure 8a), activated microglia (Figure 8b), and endothelial cells (Figure 8c). The transcriptions of ER stress signaling—ATF4 (Figure 7C) and s-XBP1 (Figure 7D)—were significantly elevated at 6 and 24 h, respectively, after ICH insult. These results indicated that ICH-induced ER stress and pro-apoptotic CHOP expression is mediated by ATF4 and sXBP1 signaling.

### 3.5. ICH-Induced Proteasome Over-Activation Exacerbates Neuroinflammation

**Figure 9:**
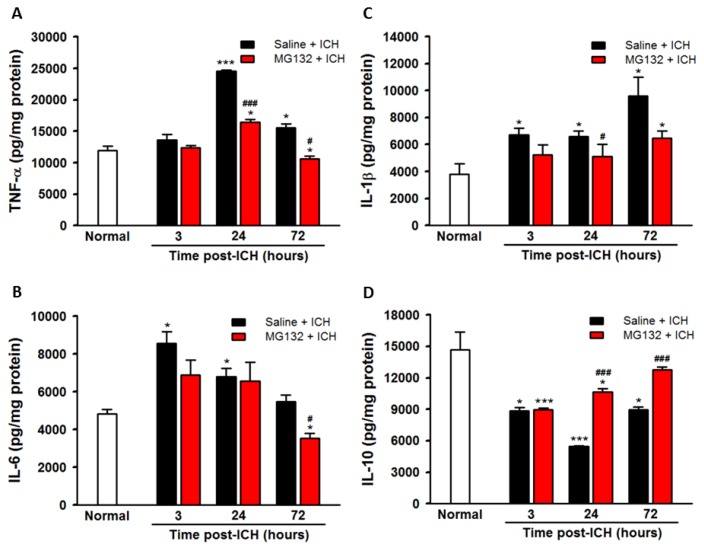
Expression levels of cytokines measured in the ipsilateral striatal tissues. The tumor necrosis factor-α (TNF-α) (**A**), interleukin (IL)-6 (**B**), IL-1β (**C**), and IL-10 (**D**) in normal, 3 h, 24 h, and 72 h post-ICH. Value are indicated by means ± SEM; n = 8 each group, * *p* < 0.05; *** *p* < 0.001; compared to normal group; ^#^ *p* < 0.05; ^###^ *p* < 0.001; compared to Saline + ICH group, respectively.

**Figure 10:**
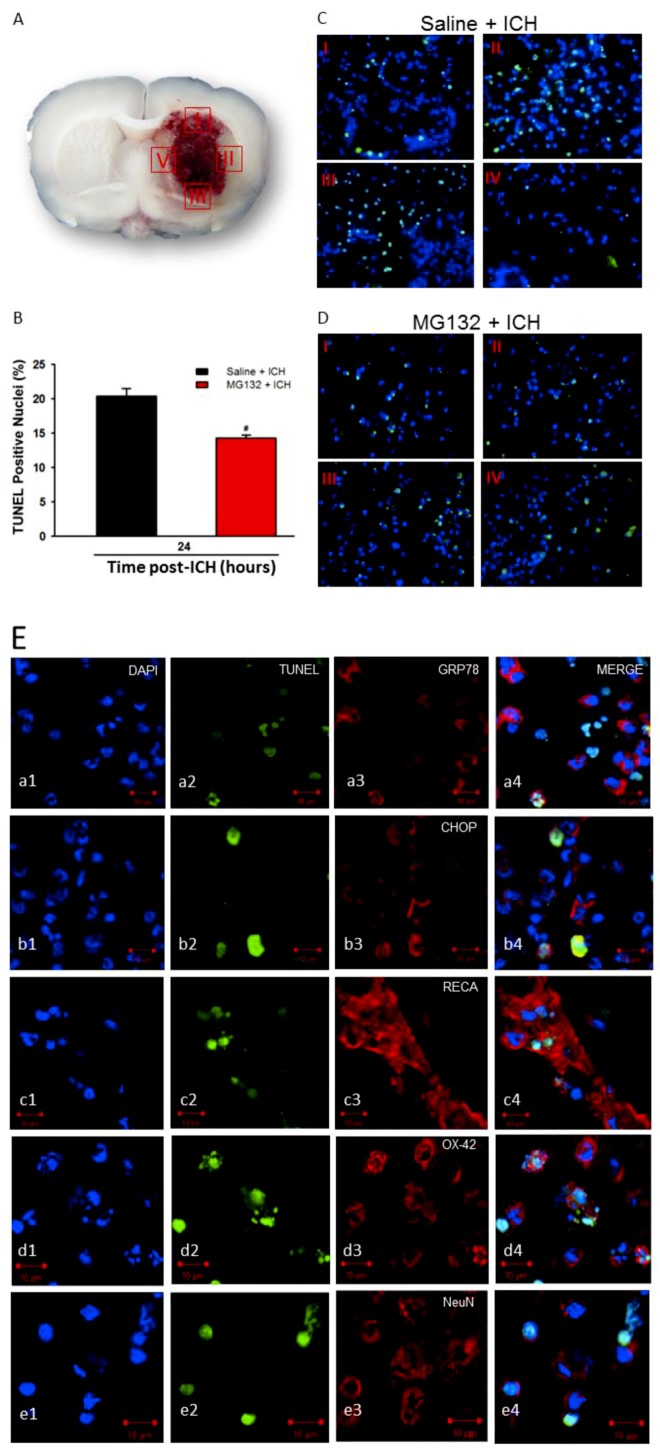
Neuronal apoptosis in hemorrhagic striatal rat at 1 day post-ICH injury. Representative TUNEL-positive staining of apoptotic cells in four regions around the perihematomal area (**A**, I–IV). Statistical measurement of apoptotic cells subjected to ICH injury in the Saline + ICH and MG132 + ICH groups (**B**), respectively. Representative TUNEL-positive image of Saline + ICH (**C**) and MG132 + ICH (**D**), respectively. Values are indicated by means ± SEM; n = 4 each group, ^#^ *p* < 0.05; as compared to the Saline + ICH group. TUNEL staining was conducted following GRP78 (**a**), CHOP (**b**), RECA (**c**), OX-42 (**d**) and NeuN (**e**) on the brain sections of the perihematomal area of ICH striatal (**E**).

In normal physiological condition, the UPR has an essential role in the selective degradation of intracellular proteins that are involved in the regulation of inflammatory processes, cell cycle regulation, and gene expression [16,20,21,23,39,40]. The proteasome has been reported to activate NF-κB through degradation of the inhibitory subunit IκB, which in turn induces inflammatory processes [41]. Our results demonstrated that the activation (phosphorylated) of NF-κB occurred rapidly after ICH injury (Figure 5D,F, Figure *p* < 0.001), which was accompanied by an early degradation of the inhibitory IκB (Figure 5D,E, *p* < 0.001) by the over-activated proteasome, especially at 3 h post-ICH (Figure 3). However, due to the huge variation of the ubiquitination of IκB, there is no statistically significant difference at 72 h post-ICH injury. The early degradation of IκB (Figure 6C,D, *p* < 0.05) decreased the expression IκB (Figure 5D,E), which unmasked the NF-κB signals (Figure 5D,F, *p* < 0.05) and induced the transcription and translation of specific pro-inflammatory genes, amplifying the inflammatory response via excessive pro-inflammatory cytokines expression, including TNF-α (Figure 9A), IL-1β (Figure 9B), and IL-6 (Figure 9C), leading to neuroinflammation and neuronal cells death (Figure 10). 

### 3.6. Apoptosis and TUNEL

The apoptotic cell death in the hemorrhagic striatum was further investigated: TUNEL-positive cells were enumerated at four specific regions (Figure 10, I–IV) around the hematomal core at 24 h post-ICH. The identity of the TUNEL-positive cells was further identified by double staining with the following antibodies: RECA, NeuN, OX-42, and GFAP, respectively (Figure 5c–e). The results demonstrated that ICH increased apoptotic cells, (Figure 10, *p* < 0.05) and apoptosis occurs in the endothelium (Figure 10c), microglia (Figure 10d), and neurons (Figure 10e), but not in astrocytes (data not shown). Most of the TUNEL-positive cells also exhibited CHOP (Figure 10b) but not GRP78 (Figure 10a), indicating that ER stress was involved in neuronal cell death. 

### 3.7. Proteasomal Inhibition Exerts Neuroprotective Effects in Alleviated Hematoma Expansion, Oxidative Stress, Body Weight Loss, and Neurological Deficits 

As compared with ICH (Saline + ICH), the intrastriatal administration of a non-selective proteasome inhibitor MG132, 3 h prior to ICH induction, exerts neuroprotective effects in reducing hematoma volume (Figure 1B), protein carbonyl content (Figure 1C), body weight loss (Figure 1D), and neurological deficits (Figure 1E) at 3, 24, and 72 h post-ICH, respectively. MG132 administration significantly blunted the ICH-induced over-activation of the proteasome during the acute phase of ICH (3 and 24 h post-ICH, Figure 3, *p* < 0.001) and rebound at 72 h post-ICH (Figure 3), which in turn significantly abolished the degradation of ubiquitinated protein, causing a massive accumulation of ubiquitinated protein peak at 3 (*p* < 0.001) hours and lasting to 72 h (*p* < 0.001) post-ICH (Figure 2A). However, this massive accumulated ubiquitinated protein was not merged with TUNEL staining in the perihematomal area (Figure S2). 

### 3.8. Proteasomal Inhibition Prevented ICH-Induced Proteasome Over-Activation, Exerts Anti-Neuroinflammation and Anti-Apoptosis 

Proteasomal inhibition by MG132 significantly restored the GRP78 expression (Figure 5A,B, *p* < 0.05 versus Saline + ICH) and thus abolished the pro-apoptotic CHOP protein expression (Figure 5A,C, *p* < 0.05 versus Saline + ICH). MG132 pre-treatment blunted the early downregulation of the IκB, restored the IκB protein level (Figure 5D,E, *p* < 0.05 versus Saline + ICH), ameliorated the activation of NF-κB (Figure 5D,F, *p* < 0.05 versus Saline + ICH) and thus, reduced pro-inflammatory cytokines (Figure 9A–C) and neuronal cells apoptosis (Figure 10 B, *p* < 0.05 versus Saline + ICH). Taken together, the inhibition of proteasome activity by MG132 ameliorated the ICH-induced over-activation of proteasome activity, and possesses neuroprotective effects through modulating the ER stress and immune responses.

## 4. Discussion

In this study, we reported for the first time that during the acute phase of ICH, ICH-induced an over-activation of the proteasome activity. This “over-zealous” proteasome activity exacerbated ER stress by the early degradation of chaperone GRP78 and increased neuroinflammation by unwanted IκB degradation, leading to activating the NFκB subset signal transduction mechanism. These detrimental impacts of the ICH-induced over-activation of the proteasome were abolished by proteasomal inhibition, exerting neuroprotective effects against ICH injury.

The pathology of ICH is dynamically complex and involves multiple cell-signaling pathways leading to neuronal cell death [2,7,37,42]. Oxidative stress is a hallmark in various neurological diseases, including ICH [4,43,44]. During ICH, the generation of excessive ROS can impair the homeostasis of ROS production, allowing for ROS to exceed the endogenous anti-oxidant capacity [4,45], leading to oxidative stress and neuronal cell death via the direct oxidation of cellular proteins, lipids, and DNA [4,45,46]. 

ER function is extremely sensitive to oxidative stress [4,18,43,44,45,47,48]. Under normal homeostasis, limited unfolded protein response (UPR) and ER stress are essential to ensure optimal cell survival [49]. However, prolonged or severe stress may overcome the UPR/ER stress functions, resulting in changes of UPR signaling from pro-survival to pro-apoptotic [4,16,18,43,45,47,50]. The overproduction of misfolded or abnormal proteins that exceed the capacity of protein degradation is an important factor caused by ER stress, which disrupts proteostasis, leading to the formation of cytotoxic protein aggregations [4,16,18,43,45,47,50]. In recent years, numerous studies have shown that the association of ER stress is important in various neurological diseases, and has been studied intensively [16,44,45,48]; however, studies have rarely reported the existence of protein aggregation or the UPR/ER stress interruption during the acute phase of hemorrhagic stroke, which may participate in the pathogenesis of diseases, including ICH. 

In the present study, during the acute phase of ICH, ICH-induced excessive oxidative stress (Figure 1) is accompanied by high levels of ubiquitination and proteasome activity (Figure 2 and Figure 3). The elevation of ubiquitin (which served as a marker of ER stress) activated the ER-associated proteasome degradation pathway in order to compensate for ER stress (relieve the misfolded/unfolded protein loading) [32,33]. However, the severity of ICH injury exceeds the capacity of UPR and consequently impaired the UPR system, and we for the first time demonstrated an over-activation of the proteasome within the hemorrhagic striatal region (Figure 3). The ICH-induced the over-activation of the proteasome, leading to the early degradation of chaperone GRP78 and IκB protein (Figure 6). This is because the function of GRP78 is to stabilize the ER stress, preventing the activation of ER stress via binding with PERK, ATF6, and IRE-1α, to maintain them in inactive forms [18,20,21,25,51]. The early degradation of GRP78 exhausted the GRP78 pools, exacerbated the ER stress by releasing ATF6 and IRE-1α, and also enabled them to transduce signals to the cytosol and nucleus. Activated IRE-1α will cleave XBP1 mRNA via inherent RNase activity, thereby yielding spliced XBP1 mRNA (sXBP1) (Figure 7C). The sXBP1 in turn will activate the pro-apoptotic transcription factor CHOP. On the other hand, activated PERK phosphorylates eukaryotic initiation factor 2α (eIF2α), which induces the activation of ATF4 that consequently induces the expression of CHOP as well. However, Zinszner et al. demonstrated that prolonged ER stress-triggered CHOP expression via PERK [52]. CHOP is implicated in programmed cell death in response to the impaired function of the endoplasmic reticulum, and the targeted disruption of CHOP delays endoplasmic reticulum stress-mediated diabetes [53]. Chop deletion reduces oxidative stress, improves β cell function, and promotes cell survival in multiple mouse models of diabetes [54]. Besides, the ICH-induced early degradation of IκB protein was observed in our experiment (Figure 6D). The regulated proteolysis of IκB is mediated by the ubiquitin–proteasome pathway [16,17]. Phosphorylation is generally believed to target IκB for ubiquitination, and ubiquitinated IκB is then selectively degraded by the 26S proteasome [16,17]. In agreement with this notion, we demonstrated that ICH-induced IκB degradation unmasked the NF-κB signals, allowing the activated NF-κB signal to translocate into the nucleus and bind to specific proinflammatory genes, amplifying the inflammatory response and increasing excessive cytokines expression, including TNF-α (Figure 9A), IL-1β (Figure 9B), and IL-6 (Figure 9C), causing neuroinflammation and neuronal cell death (Figure 10B). 

The underlying molecular mechanisms of ICH-induced neuronal cell apoptosis are diverse and complicated. Many different cell types and subcellular organelles are likely to be implicated. After ICH insult in the brains, the pro-apoptotic CHOP protein was induced and expressed within neuronal cells (Figure 8A), microglia (Figure 8B), and vascular endothelial cell (Figure 8C), but not astrocytes (data not shown). The TUNEL^-^positive apoptotic cells were not co-localized with GRP78 expression at 24 h after ICH, but they were co-localized with CHOP-positive cells (Figure 10). Taken together, we show that the GRP78 protein plays a pivotal role in neuronal cell coping capability. Degradation of the GRP78 protein at the early phase after ICH may exacerbate ICH-induced ER stress accompanied by the activation of pro-apoptotic CHOP protein expression, resulting in neuronal cell death. 

The reduction of the proteasome in ischemic stroke has been reported by several lines of evidence [35,55]. Ge et al. reported that proteasome activity was moderately decreased during the early period of reperfusion after transient brain ischemia. This downregulation of the proteasome was believed to be disassembled and aggregated [35]. However, to our knowledge, no research has studied the expression manner of proteasome activity in hemorrhagic brain injury. In addition, it is somewhat contradictory that the activity of proteasome is reduced during the course of cerebral ischemia, but exogenously administrated proteasome inhibitor possesses neuroprotection against brain injury.

Around the year 2000, injection of the proteasome inhibitor into the lateral ventricle of rats resulted in apoptotic neuronal death in various central nervous system, and also induced cell death in a different type of neurons subjected to proteasome inhibitors [56,57,58,59]. These results suggested that proteasome inhibition induced apoptotic neuronal death. In contrast with the evidence described above, the proteasome inhibitor was shown to provide neuroprotection in various models of stroke. Several general proteasome inhibitors such as MLN519, CVT-634, bortezomib, or MG132 have been demonstrated to reduce post-stroke inflammatory and protect against cerebral ischemia [60,61,62,63]. These proteasome inhibitors are believed to suppress the activation of NF-κB by stabilizing the inhibitor IκB, thereby potentially acting as anti-inflammatory agents in cerebral ischemic stroke, which was linked to both neuroprotective effects and the cell death-promoting pathway [40,55,64,65,66]. The discrepancies between the neuronal cell death and the neuroprotective effect of proteasome inhibitors in brain ischemia may be due to the different administration route and the prolonged or global blockage of the proteasome that may hamper the UPS function, which appears to be long-lasting in injury and resulting in many unwanted side effects.

In our study, MG123—a commonly used reversible proteasome inhibitor—was selected to repress the over-activation of the proteasome during the acute phase of ICH in order to avoid complete inhibition and the persistent suppression of the proteasome activity. The results demonstrated the existence of “over-activation” of the proteasome activity phenomenon. Pre-treatment with MG132 repressed ICH-induced over-activation of the proteasome (Figure 3) and ameliorated the ICH-induced oxidative stress, hematoma expansion, NF-κB activation, ER stress, and neuroinflammation, leading to the improvement of neurological deficits. 

During the acute phase of ICH, although proteasome inhibition leads to an accumulation of ubiquitin conjugates proteins (Figure 2), these ubiquitin-positive cells were not merged with TUNEL immunoreactivity, indicating that these neuronal cells possess much more tolerance with ubiquitinated proteins accumulation without any “immediate damage” effect during a short period of time (Figure S2). These findings are in agreement with the results found in neurodegenerative disorders as studied earlier, based on which neurons can resist for relatively long periods with intracellular accumulations of ubiquitinated protein [40,55,64,65,66]. 

Neurons have been reported to tolerate relatively long periods with intracellular accumulation of ubiquitinated proteins for instance in neurodegenerative disorders, but are very sensitive to damage caused by the inflammatory response [40]. The reduction of ICH injury by MG132 may be due to the net balance of their positive effects in reducing the over-zealous proteasome activity to destroy useful proteins (e.g., Chaperone GRP78 or IκB) (Figure 5 and Figure 6), which activated the NF-κB transduction mechanism, leading to neuroinflammation and negative effects in causing ubiquitinated protein accumulation (Figure 2). 

The limitation of the present study of proteasomal inhibition is that only a single type, dosage, and administration time route have been tested in our study. More data and study on their pharmacokinetic, therapeutic time window, safety profile, and toxicity are necessary before entering in a rigorous test of a clinical trial.

## 5. Conclusions

In conclusion, an ICH-induced over-activation of proteasome activity causes the robust early degradation of chaperone GRP78 and IκB protein. The degradation of chaperone GRP78 and IκB overwhelms the capability of brain cells to cope with the ER stress and the activation of the NFκB signaling, leading to the exacerbation of ER stress, proteostasis disturbance, and neuroinflammation. Uncovering the role of proteasome activity at the acute phase of ICH injury might lead to the development of an effective treatment for acute ICH injury that could be exploited therapeutically. 

## Supplementary Materials

The following are available online at https://www.mdpi.com/2073-4409/8/11/1326/s1, Figure S1: Oxidative stress (protein carbonyl content), and ubiquitin level expression in various ICH models. Oxidative stress level (protein carbonyl content, (**A**) and ubiquitinated (Ubiquitin level, B) in normal control (NC), ICH, I3: Three-fold collagenase injection volume (0.6 U in 3 microliter), and autologous blood infusion model (AB) at 3 h post-injury. Value are indicated by means ± SEM; *n* = 6 each group, * *p* < 0.05; ** *p* < 0.01; *** *p* < 0.001 as compared to normal group, respectively. Figure S2: Hematoma volume and GRP78 protein expression in various ICH models. Representative image of hemorrhagic brain sections (**A**) and statistical hematoma volume (**B**) in ICH, I3 and AB at 24 h post-ICH. Representative Western blotting images showing the GRP 78-kDa protein expression (**C**) and quantitative analyses of GRP78 protein (**D**) detected in the ipsilateral and contralateral striatum lysates at 3 h post-ICH injury. Value are indicated by means ± SEM; n = 6 each group, **p* < 0.05; ***p* < 0.01; and ****p* < 0.001 as compared to the normal group, respectively. Figure S3: Co-localization of TUNEL and Ubiquitin protein in perihematomal and core hemorrhagic striatal region at 1 day post-ICH. The yellow arrow in the merged figure indicates the co-localization of TUNEL-positive and ubiquitin-positive cells expression. The white arrow in the merged figure indicates no co-localization of TUNEL-positive and ubiquitin-positive cells expression.
