# Supplementary figures and images for: Over-Activated Proteasome Mediates Neuroinflammation on Acute Intracerebral Hemorrhage in Rats
Source: Cells. 2019 Oct 27;8(11):1326. doi: 10.3390/cells8111326 (PMC6912695; doi:10.3390/cells8111326)

Figure S1

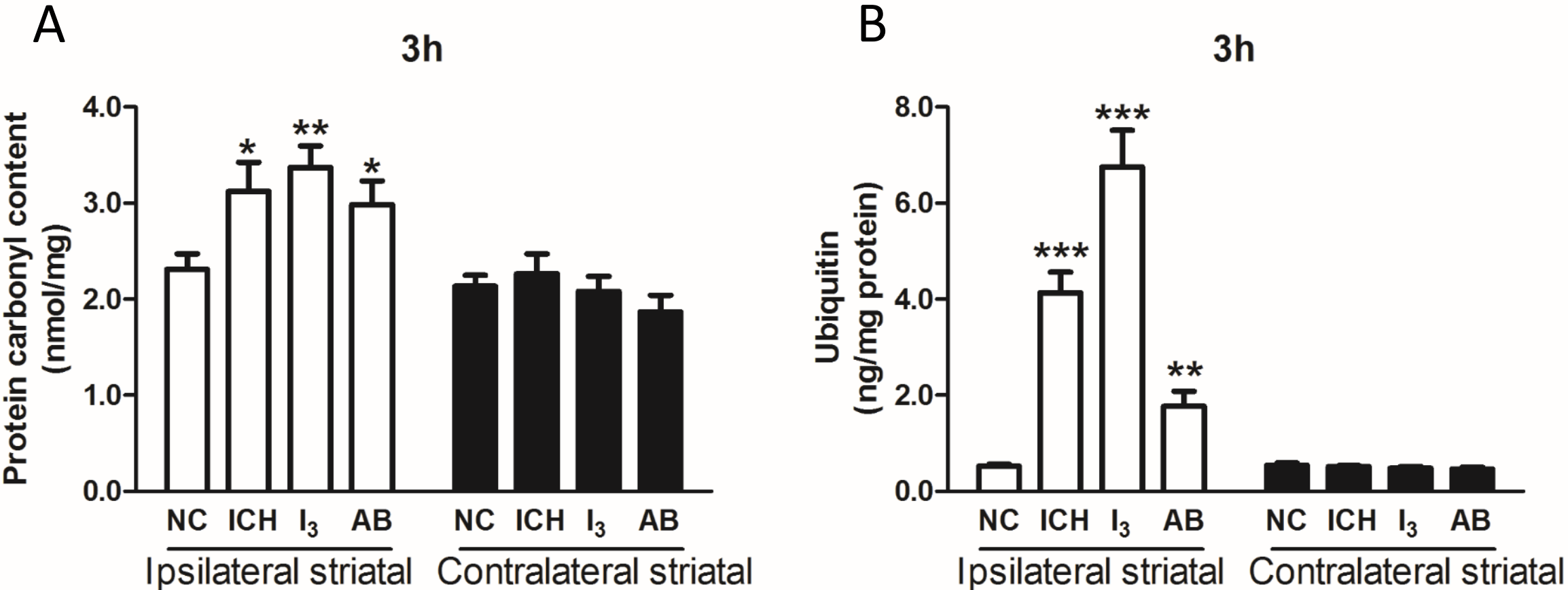

Figure S2

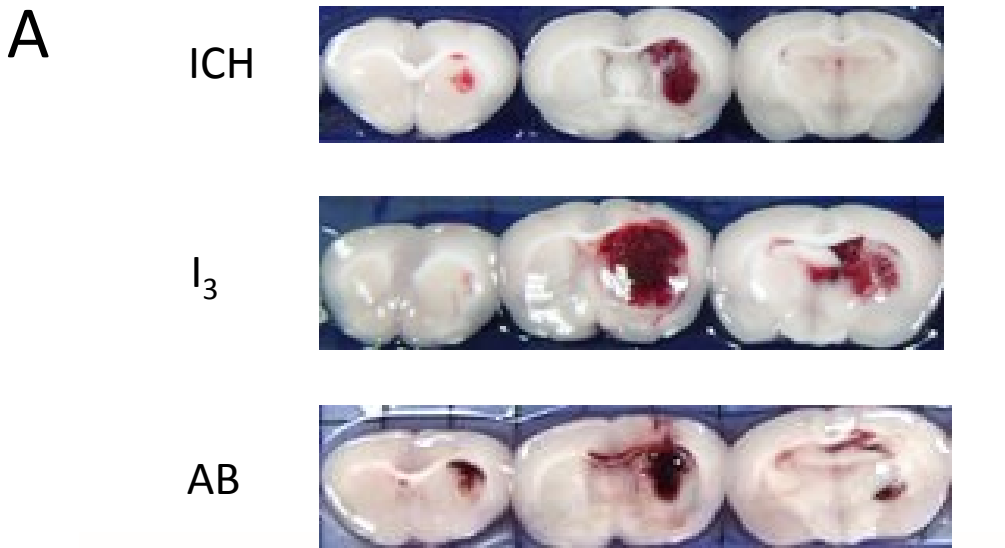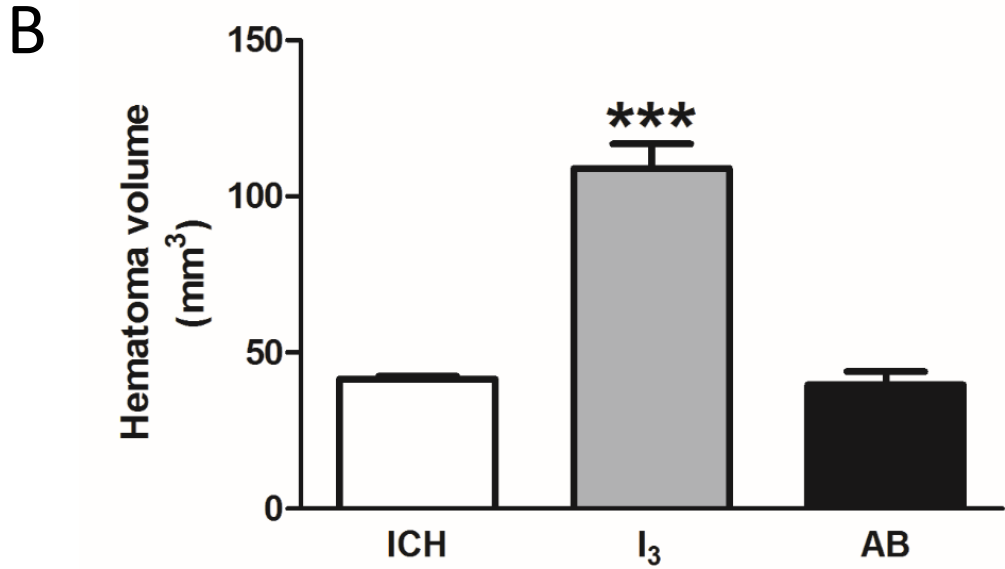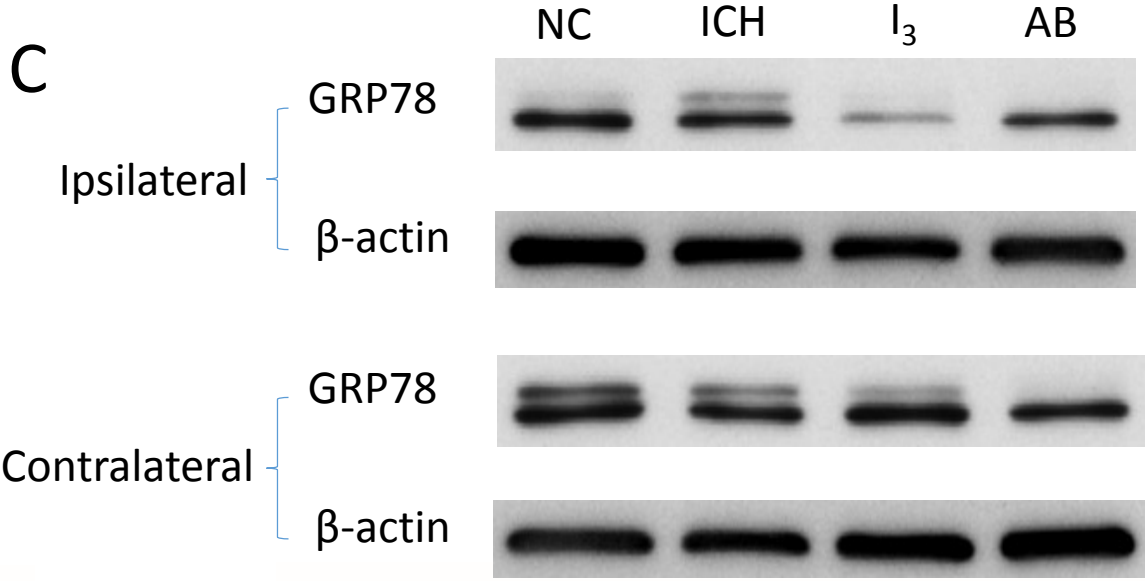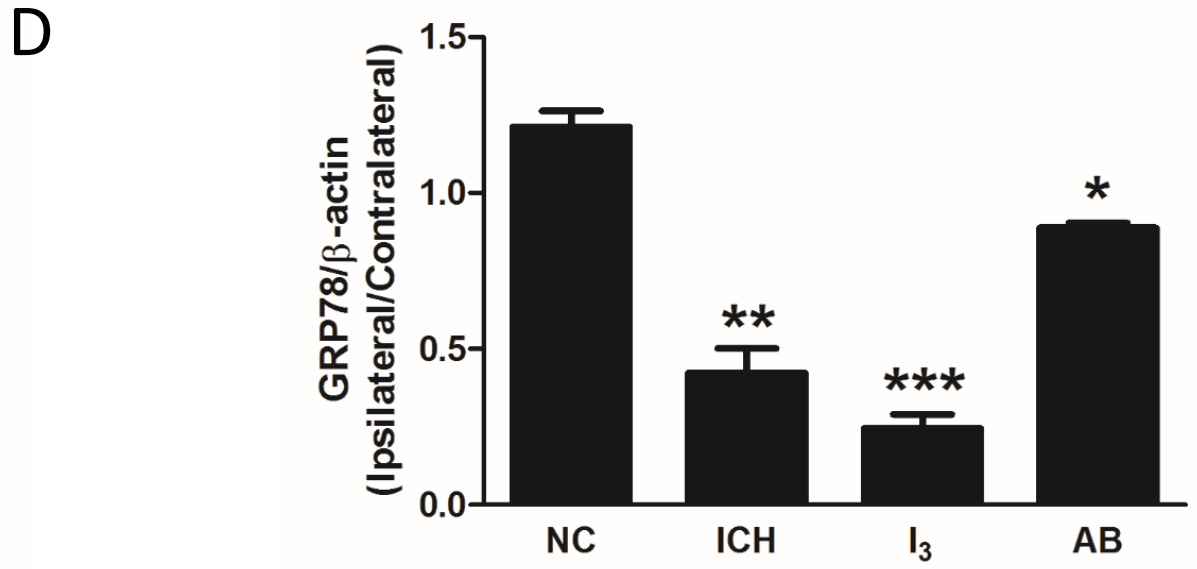

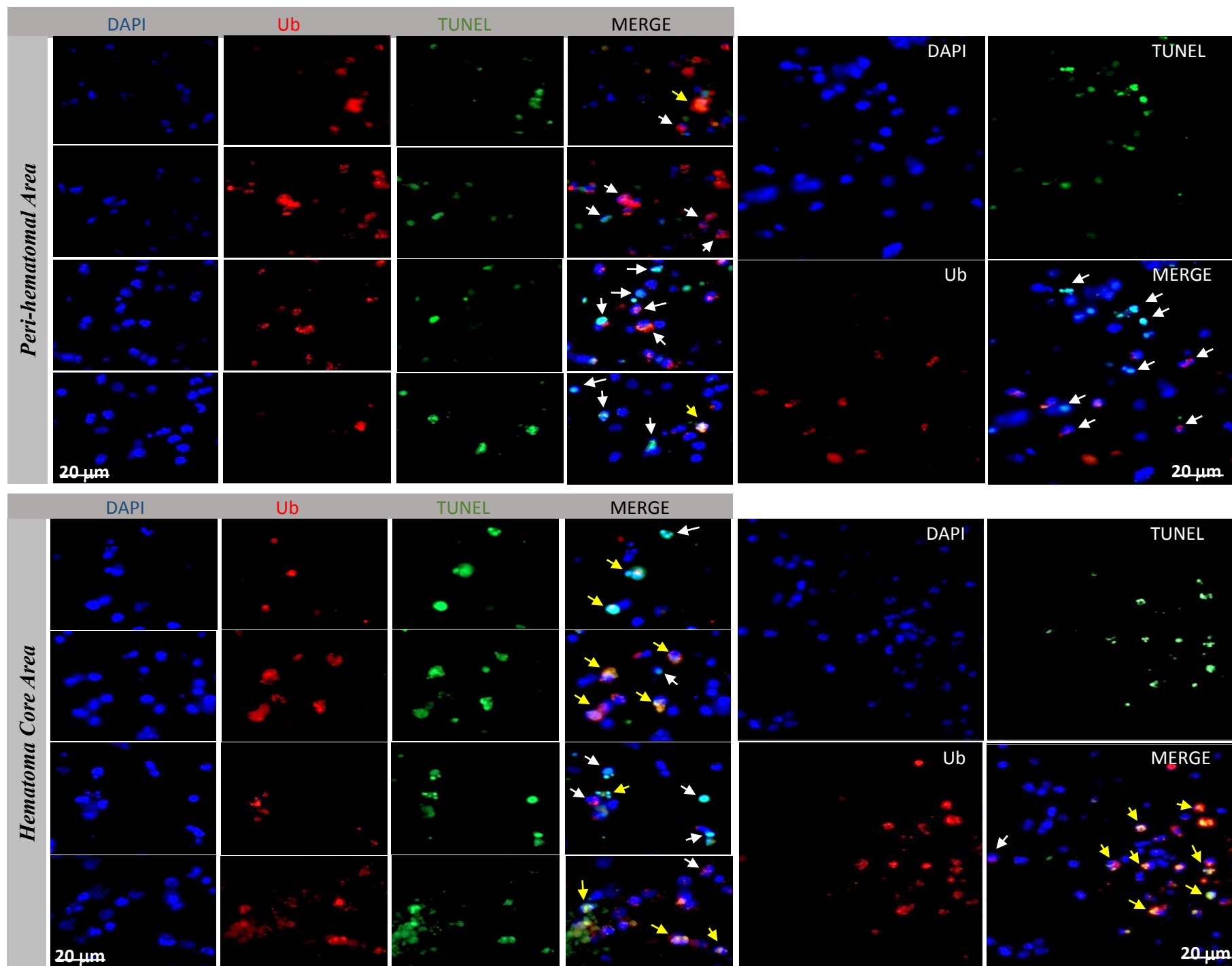

Supplement: Supplementary file 1 [file cells-08-01326-s001.pdf]
